# An International Study of Variation in Attitudes to Kidney Biopsy Practice

**DOI:** 10.2215/CJN.0000000607

**Published:** 2024-12-20

**Authors:** Michael P. Toal, Christopher J. Hill, Michael P. Quinn, Emily P. McQuarrie, Ciaran E. O’Neill, Alexander P. Maxwell

**Affiliations:** 1 Centre for Public Health, Queen’s University Belfast, Belfast, Northern Ireland; 2 Regional Centre for Nephrology and Transplantation, Belfast City Hospital, Belfast, Northern Ireland; 3 Glasgow Renal and Transplant Unit, Queen Elizabeth University Hospital, Glasgow, Scotland

**Keywords:** chronic GN, CKD, chronic renal disease, clinical nephrology, GN, health equity, diversity, and inclusion, kidney biopsy, renal biopsy, renal pathology

## Abstract

**Key Points:**

- Attitudes on kidney biopsy practice vary significantly across the world.
- Male clinicians, younger clinicians, and individuals who perform biopsies more frequently had an increased propensity to recommend a kidney biopsy.
- Kidney biopsy was most often recommended in the setting of higher proteinuria levels and preserved kidney function.

**Background:**

A kidney biopsy is an essential investigation for diagnosis but is invasive and associated with complications. Delaying or missing the opportunity to diagnose kidney disease could result in adverse patient outcomes. The aim of this study was to examine attitudes to kidney biopsy across the world.

**Methods:**

An online questionnaire for nephrologists was designed on the basis of the existing literature with input from patients. Anonymized data were collected on individual and institutional demographics, indications and contraindications for biopsy, and attitudes and barriers to access. A propensity-to-biopsy score was generated from responses, which allowed clinicians to compare their practice with international colleagues. A higher score was associated with an increased likelihood of recommending biopsy. The questionnaire was disseminated through international nephrology societies, including the National Kidney Foundation, and by social media.

**Results:**

Participants responding to the questionnaire included 1181 clinicians from 83 countries, making it the largest international study in this area to date. The propensity-to-biopsy scores were significantly different between the 13 countries with over 20 clinicians participating (*P* < 0.001) and was highest in Mexico and lowest in the Philippines. Kidney biopsy was most often recommended in patients with higher proteinuria levels and most often avoided in patients with small kidneys. An adjusted linear regression model demonstrated that a significantly higher propensity-to-biopsy score was found in male clinicians, younger clinicians, frequent performers of kidney biopsy, increased job seniority, and larger institution size (*P* = 0.05).

**Conclusions:**

Kidney biopsy practice is varied internationally and is subject to human and systemic factors. Further research is required to understand the variances behind clinical decision making.

## Visual Abstract

**Figure:**
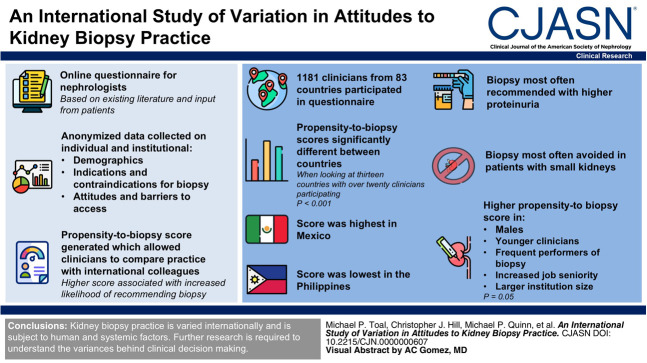

## Introduction

Kidney biopsy has been an essential tool in nephrology since the 1950s for diagnosis and guiding the treatment of kidney disease.^1,2^ Pathological scoring systems have been developed to determine prognosis on the basis of histopathological abnormalities. Although in recent years specific antibody testing has emerged as a possible noninvasive alternative for certain conditions,^3,4^ there are many diseases for which kidney biopsy remains essential for diagnosis.

Two key factors that temper enthusiasm for kidney biopsy are patient access and complications. First, kidney biopsy is an expensive pathological test requiring light microscopy and ideally additional immunofluorescence and electron microscopy. It requires ultrasound imaging, necessary equipment, appropriate facilities, and adequately trained staff. Second, the highly vascular structure of the kidney can lead to hemorrhagic complications, which, although rare, can be catastrophic.^1^ Common complications include visible hematuria, perinephric hematoma, and pain. However, severe hemorrhage can require radiological embolization or nephrectomy in around one in 300 patients, and mortality has been reported in around one in 2000 patients.^5–8^

However, these factors alone do not explain the wide variation seen in published biopsy incidence. Small studies have identified a wide range of practice patterns within individual countries, between countries, and between different socioeconomic groups.^9–12^ International guidelines recommend kidney biopsy if this information is likely to inform treatment; however, the perceived utility may be open to different interpretations.^2^ Biases and influences on clinical decision making have implications for patient care.^13^ The decision to recommend an invasive investigation may be subject to competing forces of having more diagnostic information and tolerance for risk of complications.

Personal biases and perceived utility may lead to different interpretations of when the expected benefits of biopsy outweigh potential risks.^14^ These factors have not, however, previously been systematically examined with regard to kidney biopsy practice. A standardized consensus of when to intervene and when to avoid biopsy may help eliminate inappropriate practice variations and ensure more equitable access to a timely diagnosis and treatment. This study aimed to survey a wide demographic and geographic sample of nephrologists to identify factors that may influence kidney biopsy behavior.

## Methods

Findings are described in line with the Checklist for Reporting Results of Internet E-Surveys (CHERRIES) checklist for reporting of research involving the distribution of electronic surveys.^15^

### Questionnaire Design

Clinicians eligible for inclusion were physicians from anywhere in the world who specialized in adult nephrology. A case vignette online questionnaire was designed to explore the reasoning behind clinical practice variation. The first section focused on collecting anonymized demographic details of the individual and their institution. The second section focused on decisions and attitudes (Supplemental Figure 4). Seven fictional case vignettes were presented for the physician to decide if a kidney biopsy was required for each patient on a five-point Likert scale from “definitely yes” to “definitely no.” To examine contraindications, respondents were asked to define the minimum/maximum acceptable level of laboratory results, observations, and drugs that may contraindicate biopsy. Attitudes and previously cited barriers were explored in the final part of the questionnaire.^16^

### Propensity-to-Biopsy Score

Responses to 11 questions in the Indications and Contraindications section were coded from 0 to 4. For the case vignettes, if the respondent selected “definitely yes,” this was scored as 4 points, with decreasing values for each option to “definitely no,” which was scored as 0 points. For the Contraindications section, the closest option to a normal value (*e.g*., systolic BP [SBP]=140 mm Hg) was scored as 0, whereas the selected option of proceeding to biopsy regardless of the value (*e.g*., no maximum level of SBP) was scored as 4.

This scoring system generated a propensity-to-biopsy score from 0 to 44 for each clinician who completed the entire questionnaire, with a higher score signifying the clinician was more likely to recommend biopsy. Respondents were then categorized into one of five groups on the basis of this score, and a color-coded dial was displayed on the final screen to show how their answers compared with colleagues (Supplemental Figure 1).

### Ethical Approval

Ethical approval for this project was granted by the Faculty of Medicine, Health, and Life Sciences Research Ethics Committee of Queen’s University, Belfast (Project no. MHLS 22_175), on February 15, 2023.

A statement giving consent to participate in a PhD project on kidney biopsy practice was displayed to the clinician on the first screen of the questionnaire. The provided estimate of questionnaire length was 10 minutes, and no disclosive information was collected.

### Development and Testing

Before designing the questionnaire, the research team completed a qualitative study of patient attitudes and barriers to kidney biopsy.^17^ Themes identified in this study were used to codesign the questionnaire with patient stakeholders. The research team tested the questionnaire a total of 55 times to ensure it could be completed on smartphones, tablets, or computers. The language was edited for clarity, and units were standardized for wider comprehension.

Thirty-nine clinicians in one country of the United Kingdom formed an initial pilot of the questionnaire. The mean and standard deviations from this cohort were used to define the borders between groups before wider rollout.

### Recruitment

Recruitment occurred through an open web address. Representatives from international nephrology societies, including the National Kidney Foundation, were contacted by email to ask them to distribute an anonymized link through their mailing list. The authors also shared information about the study on social media (X, formally known as Twitter) and contacted prominent nephrologists on social media platforms to ask for their assistance.

### Survey Administration

The questionnaire was hosted on Qualtrics XM^™^, which collected anonymized internet protocol (IP) addresses for regional analysis. Where clinicians had not selected their country of practice, the country was assumed to correspond to their IP address. An accurate IP address location could not be obtained for clinicians in Iran; therefore, only respondents who explicitly indicated this country were counted among national frequencies.

No incentives were offered for participation. Clinicians were recruited from August 29, 2023, through January 14, 2024. All questions were displayed in an identical order, except for the Barriers to Biopsy section, in which four options were displayed randomly.

Adaptive questioning was used to display additional questions to clinicians who selected a particular answer. The number of questions on each page ranged from one to five. The number of pages displayed to clinicians was between 17 and 19, depending on adaptive questioning. No completeness check was shown to clinicians at the end of the questionnaire; however, a back button was available to review answers.

### Response Rates

The response rates were difficult to calculate given the scale and nature of dissemination in this international study. In Northern Ireland, the Republic of Ireland, and Scotland, it is estimated that over 50% of the nephrologist workforce participated.

### Preventing Multiple Entries

The questionnaire software prevented multiple entries by the same individual and a completely automated public turing test to tell computers and humans apart feature protected against automated nonhuman respondents. Each participant was allocated a unique response ID.

### Statistical Analyses

All clinicians who met the inclusion criteria and answered one or more questions were contained in the final dataset. Clinicians who did not complete the entire questionnaire were included in the analysis of the questions they had answered; however, the propensity score was only counted if all relevant questions were answered.

The complete dataset was analyzed in Stata 17^™^ (StataCorp, College Station, TX). Aggregate results were expressed as means if normally distributed or medians if skewed. Discrete variables were reported by frequency. An unpaired *t* test was used to compare continuous variables between two groups and ANOVA with Bonferroni adjustment in the setting of three or more groups. A multiple linear regression analysis was used to examine variations in propensity score as a function of demographic characteristics, with backward elimination used to adjust for nonsignificant variables. Significance levels were tested at the 5% level (*P* < 0.05).

## Results

### Characteristics of Clinicians

A total of 1181 clinicians from 83 countries participated in the study (Supplemental Figure 2). The entire questionnaire was completed by 1077 clinicians in a median time of 5 minutes and 24 seconds. A summary of clinician characteristics is given in Table 1. Clinicians were recruited from social media and email in similar numbers. The study was open to trainees and fellows, who comprised 14.3% of the total cohort. The estimated number of biopsies performed in the previous year differed widely, and 26.8% had not performed a biopsy in the preceding year. A wide range of complications had been encountered by clinicians and a severe complication was defined as one requiring an invasive intervention or causing death.

**Table 1:** Demographic characteristics of clinicians

Parameter Selected	Frequency (%)
**Questionnaire completed**	*N*=1181
Yes	1077 (91.2)
No	104 (8.8)
**Route of completion**	*N*=1181
Email link	631 (53.4)
Social media	550 (46.6)
**Sex**	*n*=1172
Male	753 (64.3)
Female	408 (34.8)
Prefer not to say	9 (0.8)
Nonbinary/third gender	2 (0.2)
**Age, yr**	*n*=1180
20–29	30 (2.5)
30–39	442 (37.5)
40–49	327 (27.7)
50–59	251 (21.3)
60 or over	130 (11.0)
**Current job title**	*n*=1178
Trainee/fellow	168 (14.3)
Associate specialist/specialty doctor	122 (10.4)
Consultant/attending physician	733 (62.2)
Clinical director or professor	154 (13.1)
Other	1 (0.1)
**Estimated number of biopsies performed in last year**	*N*=1181
0	316 (26.8)
1–5	225 (19.1)
5–20	372 (31.5)
20–50	163 (13.8)
50 or more	105 (8.9)
**Most significant complication encountered**	*n*=1158
Non-severe	688 (59.4)^a^
*None*	116 (10.0)
*Macroscopic hematuria*	357 (30.8)
*Hematoma*	22 (1.9)
*Blood transfusion*	169 (14.6)
*Bladder dysfunction*	4 (0.4)
*Non-kidney organ injury*	6 (0.5)
*Other*	14 (1.2)
Severe	470 (40.6)^a^
*Radiological embolization*	332 (28.7)
*Emergency surgery*	10 (0.9)
*Nephrectomy*	58 (5.0)
*Death*	70 (6.0)
**Continent of Practice**	*n*=1179
Europe	405 (34.4)
North America	352 (29.9)
South America	85 (7.2)
Asia	216 (18.3)
Africa	67 (5.7)
Oceana	54 (4.6)
**Health care system**	*n*=1135
Public	658 (58.0)
Private	265 (23.4)
Public and private	202 (17.8)
Not sure	10 (0.9)
**Main place of work**	*n*=1133
Independent clinic	38 (3.4)
Rural or district general hospital	43 (3.8)
Suburban hospital	154 (13.6)
University/urban/military hospital	895 (79.0)
Other	3 (0.3)
**Typical time from referral to biopsy by radiologist**	*n*=1122
Same day	112 (10.0)
Within 1 wk	542 (48.3)
Within 1 mo	257 (22.9)
Beyond 1 mo	24 (2.1)
Unsure	187 (16.7)
**Most frequent performer of kidney biopsy at institution**	*n*=1137
Nephrologist	537 (47.2)
Radiologist	374 (32.9)
Nephrology trainee/fellow	200 (17.6)
Radiology trainee/fellow	16 (1.4)
Not sure	10 (0.9)
**Observation time after kidney biopsy**	*n*=1135
Less than 4 h	77 (6.8)
4–8 h	568 (50.0)
8–24 h	390 (34.4)
Beyond 24 h	92 (8.1)
Unsure	8 (0.7)

Partial responses included in analysis and total respondents answering each question indicated by (n) in each question.

^a^ Total number of participants indicating their most significant complication encountered as severe or non-severe.

The United States was the largest single national group, and 43 states were represented in this cohort. The four devolved nations in the United Kingdom were also represented in the second largest cumulative group. Thirteen nations had >20 clinicians included (Supplemental Table 3). Among all the respondents, 79% worked in an urban or university hospital and 58% worked in a purely publicly funded system. Waiting time for biopsy after referral to a radiologist was <1 week for 58.3% of clinicians. The usual operator specialty was nephrology in 64.8% of institutions represented; however, in 34.3% of represented institutions, most kidney biopsies were performed by a radiology specialist.

### Indications

Clinicians were asked if, in their opinion, a kidney biopsy was required in the setting of seven fictional clinical vignettes. All cases were adults with unexplained abnormalities in kidney function, reported as eGFR and/or urinary tests (hematuria or proteinuria quantified as grams per day). Four cases were a first presentation to a nephrologist, and in three, there was a dynamic change over the course of a year.

Respondents were asked to give their opinion on whether a biopsy was required on a five-point Likert scale from definitely yes, probably yes, unsure, probably not, and definitely not. Answers were coded in 0.25 increments from 1.0 to 0.0 to represent the likelihood of recommending kidney biopsy, that is, definitely yes (1.0)=100% likelihood, unsure (0.5)=50%, definitely not (0.0)=0%, and so on. Responses are summarized in Table 2 and Supplemental Table 1.

**Table 2:** Responses of clinical vignettes on indications

Patient Vignette	eGFR	Proteinuria, g	Other	Global Mean, %	Mode	Europe, %	North America, %	South America, %	Asia, %	Africa, %	Oceana, %
**First presentation**											
1	>60	4	Peripheral edema	86.8	Definitely yes	84.8	86.1	86.9	90.4	89.9	88.5
2	40	2	NVH (non-visible hematuria)	87.4	Definitely yes	84.9	86.5	91.5	91.9	86.9	89.3
3	20	2	NVH and normal kidney size	83.8	Definitely yes	82.5	81.3	84.3	89.1	85.2	87.0
4	20	2	NVH and reduced kidney size	35.5	Probably not	36.3	41.1	29.1	28.0	28.4	40.8
**Change in 1 yr**											
5	>60	0.5–2		74.9	Probably yes	69.8	73.0	82.6	82.8	83.9	71.4
6	55–40	0.5		50.5	Probably not	49.8	45.3	59.4	57.7	53.4	43.8
7	55–40	0.5–2		78.5	Probably yes	77.6	76.3	82.8	83.1	81.9	71.4

eGFR expressed as ml/min per 1.73 m^2^. Proteinuria expressed as excretion in grams per day. Mean scores in each continent expressed as a percentage. Mode refers to the most common response given for each question. NVH, nonvisible hematuria.

Questions were presented in the same order for all clinicians. Often, only one detail was changed between scenarios to determine the reasoning behind a change in answer selected. Positive indicators for kidney biopsy were higher levels of proteinuria in the setting of preserved kidney function. Negative indicators for biopsy were lower levels of proteinuria and reduced kidney size on ultrasound.

The determination of when clinicians felt the risk of kidney biopsy outweighed the benefits was explored in a section on potential contraindications, particularly relating to bleeding risk. In the first section, clinicians were presented with five options and asked for the limits of acceptable parameters to proceed to biopsy. This could be the minimum level (*e.g*., hemoglobin) or maximum level (*e.g*., SBP). The results are summarized in Table 3. There was reasonable consensus among clinicians as seen by the narrow interquartile range. Clinicians were asked to indicate the number of days a medication should be withdrawn for before proceeding to biopsy. The full range of responses was used by clinicians, which included reporting no minimum/maximum level for each parameter.

**Table 3:** Summary of responses on potential contraindications to kidney biopsy

Parameter	Global	Europe	North America	South America	Asia	Africa	Oceana
Hemoglobin—minimum acceptable, g/L	90 (80–100)	90 (80–100)	90 (80–90)	90 (90–100)	90 (80–90)	100 (80–100)	90 (80–90)
Platelet count—minimum acceptable, ×10^9^/L	100 (100)	100 (100)	100 (50–100)	100 (100)	100 (100)	100 (100–150)	100 (100)
INR—maximum acceptable	1.2 (1.2–1.4)	1.2 (1.2–1.4)	1.4 (1.2–1.4)	1.2 (1.2–1.4)	1.4 (1.2–1.4)	1.2 (1.2–1.4)	1.2 (1.2–1.4)
SBP—maximum acceptable, mm Hg	160 (140–160)	160 (150–160)	160 (140–160)	140 (140)	140 (140–160)	140 (140)	160 (160)
**No. of days held before biopsy**							
Aspirin	7 (5–7)	7 (5–7)	7 (5–7)	7 (5–7)	5 (4–7)	7 (5–7)	7 (5–7)
Clopidogrel	7 (5–7)	7 (5–7)	5 (5–7)	7 (5–7)	5 (5–7)	7 (5–7)	7 (5–7)
DOAC	3 (2–5)	3 (2–5)	3 (2–5)	4 (2–7)	5 (3–7)	5 (2–7)	3 (3–5)

Median scores in each continent with interquartile range in brackets. DOAC, direct oral anticoagulant; INR, international normalized ratio; SBP, systolic BP.

The attitudes of clinicians to kidney biopsy and potential barriers to access were explored in the final section. Clinicians were first asked to provide their opinion on three statements on a five-point scale from strongly agree to strongly disagree, which was recoded as a percentage. Finally, four potential barriers to biopsy access were displayed in a random order for clinicians to grade the significance of each from 1 to 5. This was also recoded as a percentage (Supplemental Table 2).

There was 96% agreement that biopsy was valuable to guide management and 82% agreement that it was safe. There was, however, only 68% agreement that biopsy should be performed by a nephrologist. The two most significant barriers identified by clinicians were related to the availability of a skilled operator and a suitably equipped room to perform the procedure.

**Figure 1:**
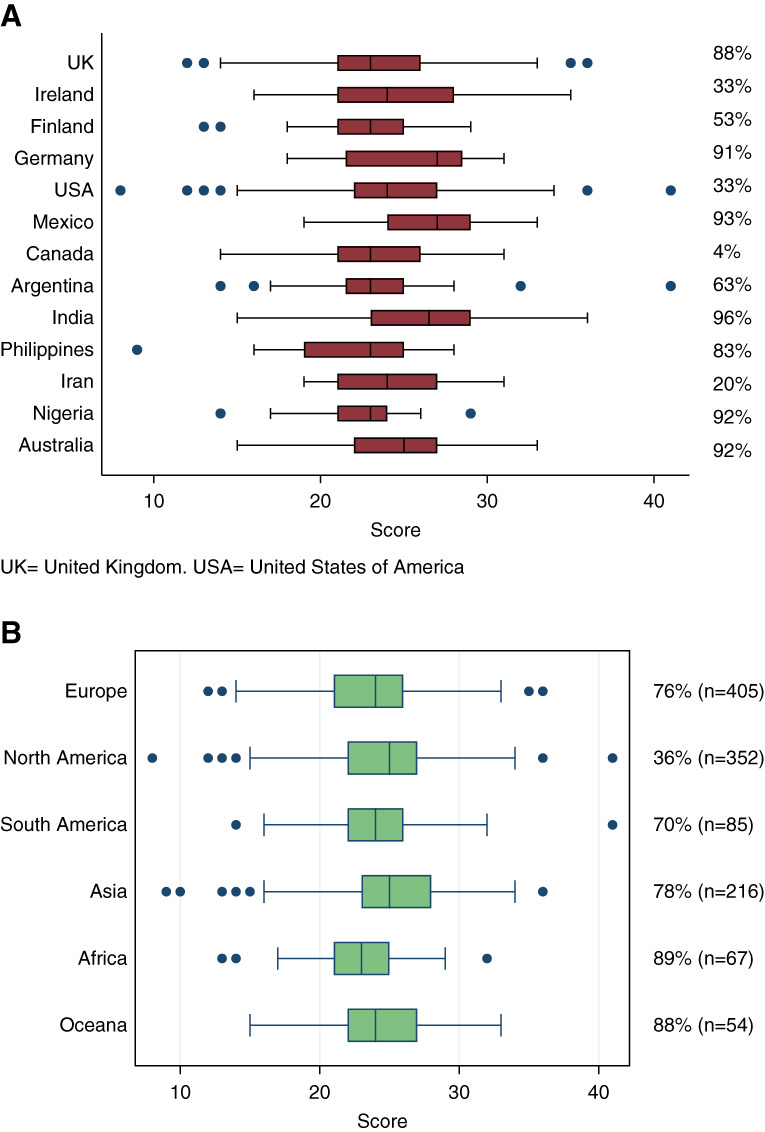
**Variation in propensity-to-biopsy scores by country and by continent.** (A) Box plot demonstrating the distribution of propensity-to-biopsy scores by country. (B) Box-plot demonstrating the distribution of propensity-to-biopsy scores by continent. Total number of participants in each continent indicated in brackets. Percentage of respondents in each country/continent indicating that the usual operator in their institution is a nephrologist. Median is indicated by a vertical line, IQR indicated by box, lower and upper adjacent values indicated by whiskers and outliers indicated by dots. IQR, interquartile range; UK, United Kingdom; US, United States.

A propensity-to-biopsy score was generated from the Indications and Contraindications sections from a scale of 0 to 44 (Supplemental Figure 3). The mean value was 24.2±4.3. When countries with over 20 clinicians were analyzed, there was a significant difference in scores (*P* < 0.001; Figure 1A). The highest propensity was demonstrated in Mexico, followed by India and Germany. The lowest propensity was noted in the Philippines, followed by Nigeria and Finland. There were significant differences in propensity scores between continents, with the highest biopsy propensity scores reported in Asia and lowest in Africa (*P* = 0.001; Figure 1B).

A multiple linear regression model was created to investigate the association between clinician demographics and propensity-to-biopsy scores. The World Bank classification of each country was used to explore the effect of national income. Five variables remained statistically significant when adjusted for covariates (Table 4). Higher propensity was demonstrated in male clinicians, younger clinicians, those who had performed biopsy more frequently in the last year, increasing job seniority, and larger institution size. No other variables remained significant, including complication severity or national income. Male clinicians had significantly higher scores in the Contraindications section than female clinicians, suggesting a greater risk tolerance (*P* < 0.001), but there was no significant difference in indications scores. Despite collecting 12 variables on demographics, the adjusted R^2^ of this model was only 5.3%; therefore, 94.7% of the variation in scores remains unexplained by these demographics.

**Table 4:** Adjusted multiple linear regression table of the effect of demographic variables on propensity-to-biopsy score

Variable	Coefficient	SEM	Significance	95% CI Lower	95% CI Upper
**Full**					
Social media response	+0.06	0.29	0.83	−0.52	+0.64
Sex (=female)	−0.73	0.29	0.01	−1.30	−0.15
Age (by increasing age group)	−0.42	0.15	0.005	−0.72	−0.13
Job title (by increasing seniority)	+0.25	0.13	0.05	−0.003	+0.49
Biopsy frequency (by increasing group)	+0.58	0.12	<0.001	+0.34	+0.81
Public health care system	−0.11	0.32	0.73	−0.75	+0.52
Institution (by increasing size)	+0.36	0.15	0.02	+0.06	+0.66
Time from referral to radiologist biopsy (faster)	+0.11	0.14	0.45	−0.17	+0.39
Observation time (by increasing length)	−0.08	0.13	0.55	−0.33	+0.17
Severe complication in past	−0.12	0.29	0.69	−0.69	+0.45
Nephrologist usual operator	−0.25	0.32	0.44	−0.88	+0.38
World Bank classification (by reducing income)	−0.05	0.21	0.81	−0.47	+0.37
**Limited**					
Sex (female)	−0.77	0.28	0.007	−1.33	−0.21
Age (by increasing age group)	−0.45	0.14	0.002	−0.73	−0.17
Job title (by increasing seniority)	+0.24	0.12	0.05	+0.002	+0.47
Biopsy frequency (by increasing group)	+0.54	0.11	<0.001	+0.33	+0.75
Institution (by increasing size)	+0.30	0.14	0.03	+0.02	+0.58

*N*=1022, F=12.4. Adjusted R^2^=0.0527. CI, confidence interval.

Participants reported variations in the usual operator for kidney biopsy at their institution, with 34% of clinicians reporting that most procedures are performed by a radiologist. Participants from these institutions reported no significant difference in propensity scores compared with colleagues from institutions where nephrologists did most of the kidney biopsies; however, for multiple clinical vignettes, a significantly higher propensity was reported by clinicians from a predominantly nephrologist-led service (Table 5). Observation times after biopsy were typically shorter in radiology-led units and public institutions.

**Table 5:** Questionnaire responses given divided by the usual operator in each institution

Parameter Selected	Nephrologist Usual Operator	Radiologist Usual Operator	Significance
Case 1	88%	84%	<0.001
Case 2	88%	85%	0.01
Case 3	85%	81%	0.005
Case 4	35%	35%	0.96
Case 5	75%	73%	0.18
Case 6	53%	46%	<0.001
Case 7	79%	77%	0.08
Hemoglobin	86	84	0.07
Platelets	98	94	0.007
INR	1.31	1.33	0.20
SBP	153	155	0.02
Aspirin	5.6	5.7	0.46
Clopidogrel	6.1	5.9	0.03
DOAC	3.9	3.9	0.98
**Annual biopsies performed, No. (%)**			<0.001
0	160 (22)	144 (37)	
1–5	131 (18)	73 (19)	
5–20	219 (30)	138 (35)	
20–50	135 (18)	27 (7)	
50+	92 (12)	8 (2)	
**Observation time, No. (%)**			<0.001
<4 h	30 (4)	45 (12)	
4–8 h	369 (50)	198 (51)	
8–24 h	270 (37)	116 (30)	
>24 h	66 (9)	25 (6)	
Unsure	1 (0)	4 (1)	
Severe complication	39%	45%	0.06
Propensity score	24.33	24.01	0.26

DOAC, direct oral anticoagulant; INR, international normalized ratio; SBP, systolic BP.

## Discussion

This study is the first worldwide survey of attitudes to the utility and practice of kidney biopsy. This has revealed wide variations in opinions around kidney biopsy practice, which are unlikely to be explained simply by inequitable access to health care resources and was not influenced by experience of complications.^18–20^ National cohesion may be explained by successive training of doctors in that system, but even within countries, there was significant variation as shown in Figure 1A. There does not seem to be a gradient in biopsy propensity by World Bank income classification, although this could be due to ascertainment bias in respondents from predominantly high-income countries. Studies examining risk aversion in demographic groups generally have shown mixed findings with no consistent patterns related to age or sex.^21–23^ There are likely many conscious and unconscious biases on clinician decision making and uncertainty may be alleviated by requesting more investigations.^24^ In this study, however, younger male doctors were more likely to recommend kidney biopsy, as were those who undertake biopsy frequently. Job seniority and institution size were also associated with modestly increased propensity scores. The finding that practice was not affected by the history of a severe complication was unexpected, although that probably reflects clinical experience. Two significant patterns seem to emerge from the data on indications to undertake kidney biopsy: first, that the decision to biopsy is driven more by urinary abnormalities than by reduced eGFR. Second, the perceived risk-benefit balance is significantly changed when the kidneys are small. This finding could be interpreted in two ways, that biopsies of small kidneys may be of limited utility and/or that there is an increased perceived risk of a complication.

The largest preceding study of biopsy practice was on 220 Japanese facility directors, who reported more liberal contraindications to biopsy than demonstrated in this study, with acceptable limits of hemoglobin, platelets, international normalized ratio, and BP that were much more abnormal.^12^ The contraindications described in this international study are similar to the guidelines of the French National Authority for Health (FNAH), which were published subsequent to our recruitment period.^25^ Notable exceptions to these similarities were the international normalized ratio (<1.5) and aspirin (continued or held for 5 days) thresholds, for which the FNAH recommended less stringent limits than were described in this study. The FNAH guidelines also acknowledge the limited evidence base for established biopsy practices with many recommendations on the basis of expert agreement rather than evidence from clinical trials.^25^

The rapid pace of developments in precision medicine and artificial intelligence leads to the question of how the role of kidney biopsy may change as we move through the 21st century.^26,27^ Highly specific, noninvasive antibody testing has already revolutionized how rapidly progressive GN and membranous nephropathy are diagnosed and monitored.^4,28^ If further developments continue on this front, kidney biopsy and its consequences may be partly superseded by novel techniques—the so-called liquid biopsy. However, only a limited number of biomarkers are currently available. In this questionnaire, clinicians indicated that at present kidney biopsy remains a cornerstone of care in guiding management. The role of radiologists in kidney biopsy practice remains essential in certain regions of the world, with around one third of biopsies performed by radiology in this survey, but there is significant debate as to which specialty should continue to perform this procedure.^16,29–31^

This study has some limitations. As is inevitable in all questionnaire-based research, clinicians were not randomly selected and it is possible that individuals who participated are different from those who declined. This is mitigated in several countries within the United Kingdom and the Republic of Ireland with large participation rates that captured high proportions of the eligible population. Second, the clinical vignettes cannot fully capture all the nuances and considerations that go into deciding to perform a kidney biopsy. This was a necessary step to ensure the accessibility of the study. All questions were open to potential misinterpretation, and it is possible that some inaccurate responses were given in error. Finally, the reasoning behind these decisions is not explored in depth, and this would require further qualitative studies.

The study also has significant strengths. Previous literature on kidney biopsy practice is limited to smaller studies from single countries. The large number of clinicians from countries across the world makes this the most representative study of its kind. The accessibility of the study facilitated the high uptake. Internet-based sources and social media platforms were used effectively to reach clinicians across the world. Previous biopsy studies have not sought to seek reasons behind the differences in clinical decisions, and this study uses regression models to explore these questions.

This study raises other questions which require future research, particularly looking at why individuals make these decisions, which may change over time as kidney biopsy practice is impacted by new developments in precision medicine. In-depth interviews with nephrologists may explain why they are making these decisions and what key information is required.

There is marked variability in attitudes to kidney biopsy practice across the world. Increased propensity to biopsy was demonstrated in male clinicians, younger clinicians, and individuals who perform biopsy more frequently. Job seniority and institution size also demonstrated modestly increased propensity scores. A history of a severe complication resulting from this procedure did not significantly change attitudes on the practice. This suggests that individual variation more than system- or country-based variation is responsible for differences seen.

Equitable access to kidney biopsy is essential for timely diagnosis and management of kidney disease. Further research is required to better understand the wide variations in practice identified in this study.

## Supplementary Material

## Data Availability

Partial restrictions to the data and/or materials apply. Data utilized in this study is not available on a public repository in line with recommendations from the approving Research Ethics Committee and to ensure the anonymity of participants is protected. However, data sharing can be considered upon reasonable request by contacting the corresponding author.

## Supplemental Material

Supplemental Figure 1. Color-coded dial.

Supplemental Figure 2A. Map of countries represented in the study.

Supplemental Figure 2B. Map of countries by national participant frequency.

Supplemental Figure 3A. Histogram of propensity-to-biopsy scores.

Supplemental Figure 3B. Histogram of propensity-to-biopsy scores by continent.

Supplemental Table 1. Indications for biopsy responses.

Supplemental Table 2. Attitudes and barriers to kidney biopsy.

Supplemental Table 3. Full list of participating countries.

Supplemental Figure 4. Electronic questionnaire instrument.

## References

[1] Hogan JJ Mocanu M Berns JS. The native kidney biopsy: update and evidence for best practice. Clin J Am Soc Nephrol. 2016;11(2):354–362. doi:10.2215/CJN.05750515 26339068 PMC4741037

[2] Adler SG Barratt J Bridoux F, .; Kidney Disease Improving Global Outcomes (KDIGO) Glomerular Diseases Work Group. KDIGO 2021 clinical practice guideline for the management of glomerular diseases. Kidney Int. 2021;100(4S):S1–S276. doi:10.1016/j.kint.2021.05.021 34556256

[3] Roberts ISD Cook HT Troyanov S, .; Working Group of the International IgA Nephropathy Network and the Renal Pathology Society. The Oxford classification of IgA nephropathy: pathology definitions, correlations, and reproducibility. Kidney Int. 2009;76(5):546–556. doi:10.1038/ki.2009.168 19571790

[4] Beck LH Bonegio RG Lambeau G, . M-type phospholipase A 2 receptor as target antigen in idiopathic membranous nephropathy. N Engl J Med. 2009;361(1):11–21. doi:10.1056/NEJMoa0810457 19571279 PMC2762083

[5] Waldo B Korbet SM Freimanis MG Lewis EJ. The value of post-biopsy ultrasound in predicting complications after percutaneous renal biopsy of native kidneys. Nephrol Dial Transplant. 2009;24(8):2433–2439. doi:10.1093/ndt/gfp073 19246472

[6] Bandari J Fuller TW Turner Іі RM D’Agostino LA. Renal biopsy for medical renal disease: indications and contraindications. Can J Urol. 2016;23(1):8121–8126. PMID: 26892051 26892051

[7] Poggio ED McClelland RL Blank KN, .; Kidney Precision Medicine Project. Systematic review and meta-analysis of native kidney biopsy complications. Clin J Am Soc Nephrol. 2020;15(11):1595–1602. doi:10.2215/CJN.04710420 33060160 PMC7646247

[8] Whittier WL Korbet SM. Timing of complications in percutaneous renal biopsy. J Am Soc Nephrol. 2004;15(1):142–147. doi:10.1097/01.ASN.0000102472.37947.14 14694166

[9] McQuarrie EP Mackinnon B McNeice V Fox JG Geddes CC. The incidence of biopsy-proven IgA nephropathy is associated with multiple socioeconomic deprivation. Kidney Int. 2014;85(1):198–203. doi:10.1038/ki.2013.329 24025641

[10] McQuarrie EP MacKinnon B Young B, .; Scottish Renal Biopsy Registry. Centre variation in incidence, indication and diagnosis of adult native renal biopsy in Scotland. Nephrol Dial Transplant. 2009;24(5):1524–1528. doi:10.1093/ndt/gfn677 19074409

[11] Burke JP Pham T May S, . Kidney biopsy practice amongst Australasian nephrologists. BMC Nephrol. 2021;22(1):291. doi:10.1186/s12882-021-02505-9 34445981 PMC8390249

[12] Kawaguchi T Nagasawsa T Tsuruya K, .; Committee of Practical Guide for Kidney Biopsy 2019. A nationwide survey on clinical practice patterns and bleeding complications of percutaneous native kidney biopsy in Japan. Clin Exp Nephrol. 2020;24(5):389–401. doi:10.1007/s10157-020-01869-w 32189101 PMC7174253

[13] Nouhi M Hadian M Jahangiri R Hakimzadeh M Gray S Olyaeemanesh A. The economic consequences of practice style variation in providing medical interventions: a systematic review of the literature. J Educ Health Promot. 2019;8(119):119. doi:10.4103/jehp.jehp_386_18 31334271 PMC6615132

[14] Djulbegovic B van den Ende J Hamm RM Mayrhofer T Hozo I Pauker SG.; International Threshold Working Group (ITWG). When is rational to order a diagnostic test, or prescribe treatment: the threshold model as an explanation of practice variation. Eur J Clin Invest. 2015;45(5):485–493. doi:10.1111/eci.12421 25675907

[15] Eysenbach G. Improving the quality of web surveys: the checklist for reporting results of Internet E-surveys (CHERRIES). J Med Internet Res. 2004;6(3):e34. doi:10.2196/jmir.6.3.e34 15471760 PMC1550605

[16] Yuan CM Nee R Little DJ, .; Nephrology Education Research and Development Consortium (NERDC). Survey of kidney biopsy clinical practice and training in the United States. Clin J Am Soc Nephrol. 2018;13(5):718–725. doi:10.2215/CJN.13471217 29669819 PMC5968891

[17] Toal M Raynor M McKeaveney C, . ‘Was my kidney biopsy worth it?’–A qualitative phenomenological study of patient experiences and perceived barriers to kidney biopsy. PLoS One. 2024;19(9):e0310358. doi:10.1371/journal.pone.0310358 39259730 PMC11389898

[18] Liyanage T Ninomiya T Jha V, . Worldwide access to treatment for end-stage kidney disease: a systematic review. Lancet. 2015;385(9981):1975–1982. doi:10.1016/S0140-6736(14)61601-9 25777665

[19] Okoye O Mamven M. Global dialysis perspective: Nigeria. Kidney360. 2022;3(9):1607–1610. doi:10.34067/KID.0002312022 36245658 PMC9528372

[20] Kumashie DD Tiwari R Hassen M Chikte UME Davids MR. Trends in the nephrologist workforce in South Africa (2002–2017) and forecasting for 2030. PLoS One. 2021;16(8):e0255903. doi:10.1371/journal.pone.0255903 34383826 PMC8360377

[21] Franks P Williams GC Zwanziger J Mooney C Sorbero M. Why do physicians vary so widely in their referral rates? J Gen Intern Med. 2000;15(3):163–168. doi:10.1046/j.1525-1497.2000.04079.x 10718896 PMC1495354

[22] Hall KH. Reviewing intuitive decision-making and uncertainty: the implications for medical education. Med Educ. 2002;36(3):216–224. doi:10.1046/j.1365-2923.2002.01140.x 11879511

[23] Sacks GD Dawes AJ Tsugawa Y, . The association between risk aversion of surgeons and their clinical decision-making. J Surg Res. 2021;268:232–243. doi:10.1016/j.jss.2021.06.056 34371282

[24] Kassirer J. Our stubborn quest for diagnostic certainty. A cause of excessive testing. New Engl J Med. 1989;320(22):1489–1491. doi:10.1056/NEJM198906013202211 2497349

[25] de Laforcade L Bobot M Boffa JJ, . Kidney biopsy for the diagnosis and treatment of kidney diseases. Recommendations from the French speaking Society of Nephrology (SFNDT) and French National Authority for Health (HAS) 2022. Nephrol Ther. 2024;20(1):61–80. doi:10.1684/ndt.2024.68 38379375

[26] Najjar R. Redefining radiology: a review of artificial intelligence integration in medical imaging. Diagnostics. 2023;13(17):2760. doi:10.3390/diagnostics13172760 37685300 PMC10487271

[27] Ayers JW Poliak A Dredze M, . Comparing physician and artificial intelligence chatbot responses to patient questions posted to a public social media Forum. JAMA Intern Med. 2023;183(6):589–596. doi:10.1001/jamainternmed.2023.1838 37115527 PMC10148230

[28] Chevet B Cornec D Casal Moura M, . Diagnosing and treating ANCA-Associated vasculitis: an updated review for clinical practice. Rheumatology. 2023;62(5):1787–1803. doi:10.1093/rheumatology/keac623 36315063

[29] Nascimento MM Chula D Campos R Nascimento D Riella MC. Interventional nephrology in Brazil: current and future status. Semin Dial. 2006;19(2):172–175. doi:10.1111/j.1525-139X.2006.00146.x 16551298

[30] Berns JS. Training nephrology fellows in temporary hemodialysis catheter placement and kidney biopsies is needed and should be required. Clin J Am Soc Nephrol. 2018;13(7):1099–1101. doi:10.2215/CJN.00040118 29907618 PMC6032578

[31] Amodu A Porteny T Schmidt IM Ladin K Waikar SS. Nephrologists’ attitudes toward native kidney biopsy: a qualitative study. Kidney Med. 2021;3(6):1022–1031. doi:10.1016/j.xkme.2021.06.014 34939011 PMC8664729

