# Supplementary material for: An International Study of Variation in Attitudes to Kidney Biopsy Practice
Source: Clin J Am Soc Nephrol. 2024 Dec 20;20(3):377–86. doi: 10.2215/CJN.0000000607 (PMC11906007; doi:10.2215/CJN.0000000607)
Supplement: Supplementary file 1 [file cjasn-20-377-s001.pdf]

ASN American Society of Nephrology | JASN Journal of the American Society of Nephrology | KIDNEY360 Accessing Our World From Every Angle | CJASN Clinical Journal of the American Society of Nephrology

ASN Journal Disclosure Form

As per ASN journal policy, I have disclosed any financial relationships or commitments I have held in the past 36 months as included below. I have listed my Current Employer below to indicate there is a relationship requiring disclosure. If no relationship exists, my Current Employer is not listed.

C. Hill reports the following:
Employer: Belfast Health and Social Care Trust; and Ownership Interest: Lloyd's Banking Group PLC - stock; Tenaya Therapeutic - stock; Royal London - corporate bond;.

I understand that the information above will be published within the journal article, if accepted, and that failure to comply and/or to accurately and completely report the potential financial conflicts of interest could lead to the following: 1) Prior to publication, article rejection, or 2) Post-publication, sanctions ranging from, but not limited to, issuing a correction, reporting the inaccurate information to the authors' institution, banning authors from submitting work to ASN journals for varying lengths of time, and/or retraction of the published work.

Name: Christopher Hill
Manuscript ID: CJASN-2024-001102R1
Manuscript Title: An international study of variation in attitudes to kidney biopsy practice
Date of Completion: October 8, 2024
Disclosure Updated Date: October 8, 2024

ASN American Society of Nephrology | JASN Journal of the American Society of Nephrology | KIDNEY360 Accessing Our World From Every Angle | CJASN Clinical Journal of the American Society of Nephrology

ASN Journal Disclosure Form

As per ASN journal policy, I have disclosed any financial relationships or commitments I have held in the past 36 months as included below. I have listed my Current Employer below to indicate there is a relationship requiring disclosure. If no relationship exists, my Current Employer is not listed.

A. Maxwell has nothing to disclose.

I understand that the information above will be published within the journal article, if accepted, and that failure to comply and/or to accurately and completely report the potential financial conflicts of interest could lead to the following: 1) Prior to publication, article rejection, or 2) Post-publication, sanctions ranging from, but not limited to, issuing a correction, reporting the inaccurate information to the authors' institution, banning authors from submitting work to ASN journals for varying lengths of time, and/or retraction of the published work.

Name: Alexander P. Maxwell
Manuscript ID: CJASN-2024-001102R1
Manuscript Title: An international study of variation in attitudes to kidney biopsy practice
Date of Completion: October 7, 2024
Disclosure Updated Date: September 28, 2024

ASN American Society of Nephrology | JASN Journal of the American Society of Nephrology | KIDNEY360 Accessing Our World From Every Angle | CJASN Clinical Journal of the American Society of Nephrology

ASN Journal Disclosure Form

As per ASN journal policy, I have disclosed any financial relationships or commitments I have held in the past 36 months as included below. I have listed my Current Employer below to indicate there is a relationship requiring disclosure. If no relationship exists, my Current Employer is not listed.

E. Mcquarrie reports the following:
Employer: NHS GGC; and Honoraria: AstraZeneca; Vifor.

I understand that the information above will be published within the journal article, if accepted, and that failure to comply and/or to accurately and completely report the potential financial conflicts of interest could lead to the following: 1) Prior to publication, article rejection, or 2) Post-publication, sanctions ranging from, but not limited to, issuing a correction, reporting the inaccurate information to the authors' institution, banning authors from submitting work to ASN journals for varying lengths of time, and/or retraction of the published work.

Name: Emily Mcquarrie
Manuscript ID: CJASN-2024-001102R1
Manuscript Title: An international study of variation in attitudes to kidney biopsy practice
Date of Completion: October 8, 2024
Disclosure Updated Date: October 8, 2024

ASN
American Society of Nephrology
JASN
Journal of the American Society of Nephrology
KIDNEY360
Accessing Our World From Every Angle
CJASN
Clinical Journal of the American Society of Nephrology

ASN Journal Disclosure Form

As per ASN journal policy, I have disclosed any financial relationships or commitments I have held in the past 36 months as included below. I have listed my Current Employer below to indicate there is a relationship requiring disclosure. If no relationship exists, my Current Employer is not listed.

C. O'Neill reports the following:
Employer: Queen's University Belfast; Consultancy: CSL Seqirus; Research Funding: Astra Zeneca; Advisory or Leadership Role: CSL Sequris; Speakers Bureau: CSL Seqirus; and Other Interests or Relationships: Medtronic and Pfizer have both provided sponsorship to help run a masterclass in health economics.

I understand that the information above will be published within the journal article, if accepted, and that failure to comply and/or to accurately and completely report the potential financial conflicts of interest could lead to the following: 1) Prior to publication, article rejection, or 2) Post-publication, sanctions ranging from, but not limited to, issuing a correction, reporting the inaccurate information to the authors' institution, banning authors from submitting work to ASN journals for varying lengths of time, and/or retraction of the published work.

Name: Ciaran O'Neill
Manuscript ID: CJASN-2024-001102R1
Manuscript Title: An international study of variation in attitudes to kidney biopsy practice
Date of Completion: October 8, 2024
Disclosure Updated Date: October 8, 2024

ASN
American Society of Nephrology
JASN
Journal of the American Society of Nephrology
KIDNEY360
Accessing Our World From Every Angle
CJASN
Clinical Journal of the American Society of Nephrology

ASN Journal Disclosure Form

As per ASN journal policy, I have disclosed any financial relationships or commitments I have held in the past 36 months as included below. I have listed my Current Employer below to indicate there is a relationship requiring disclosure. If no relationship exists, my Current Employer is not listed.

M. Quinn reports the following:
Employer: Queens University Belfast; Consultancy: British Telecom; and Advisory or Leadership Role: Clinical Advisory Board BT ; Founder Round Safely.

I understand that the information above will be published within the journal article, if accepted, and that failure to comply and/or to accurately and completely report the potential financial conflicts of interest could lead to the following: 1) Prior to publication, article rejection, or 2) Post-publication, sanctions ranging from, but not limited to, issuing a correction, reporting the inaccurate information to the authors' institution, banning authors from submitting work to ASN journals for varying lengths of time, and/or retraction of the published work.

Name: Michael P. Quinn
Manuscript ID: CJASN-2024-001102R1
Manuscript Title: An International study of variation in attitudes to kidney biopsy practice
Date of Completion: October 7, 2024
Disclosure Updated Date: April 26, 2024

ASN American Society of Nephrology
JASN Journal of the American Society of Nephrology
KIDNEY360 Accessing Our World From Every Angle
CJASN Clinical Journal of the American Society of Nephrology

ASN Journal Disclosure Form

As per ASN journal policy, I have disclosed any financial relationships or commitments I have held in the past 36 months as included below. I have listed my Current Employer below to indicate there is a relationship requiring disclosure. If no relationship exists, my Current Employer is not listed.

M. Toal reports the following:
Employer: Belfast City Hospital

I understand that the information above will be published within the journal article, if accepted, and that failure to comply and/or to accurately and completely report the potential financial conflicts of interest could lead to the following: 1) Prior to publication, article rejection, or 2) Post-publication, sanctions ranging from, but not limited to, issuing a correction, reporting the inaccurate information to the authors' institution, banning authors from submitting work to ASN journals for varying lengths of time, and/or retraction of the published work.

Name: Michael Toal
Manuscript ID: CJASN-2024-001102R1
Manuscript Title: An international study of variation in attitudes to kidney biopsy practice
Date of Completion: October 8, 2024
Disclosure Updated Date: April 26, 2024
