# Supplementary material for: An International Study of Variation in Attitudes to Kidney Biopsy Practice
Source: Clin J Am Soc Nephrol. 2024 Dec 20;20(3):377–86. doi: 10.2215/CJN.0000000607 (PMC11906007; doi:10.2215/CJN.0000000607)
Supplement: Supplementary file 2 [file cjasn-20-377-s002.pdf]

Supplement: An international study of variation in attitudes to kidney biopsy practice

Contents

Figure S1: Color-coded dial 2
Figure S2A: Map of countries represented in study. 2
Figure. S2B. Map of countries by national participant frequency 3
Figure S3A: Histogram of propensity-to-biopsy scores. 4
Figure S3B: Histogram of propensity-to-biopsy scores by continent 5
Table S1: Indications for biopsy responses 6
Table S2: Attitudes and barriers to kidney biopsy 6
Table S3: Full list of participating countries 6
Figure S4: Electronic questionnaire instrument 8

Figure S1: Color-coded dial

Fig. 1. Color-coded dial displayed to clinicians on completion of the questionnaire, indicating their score in comparison with colleagues with an explanatory message. Dark green indicated most likely to recommend kidney biopsy compared to other clinicians. Red indicated least likely to recommend kidney biopsy.

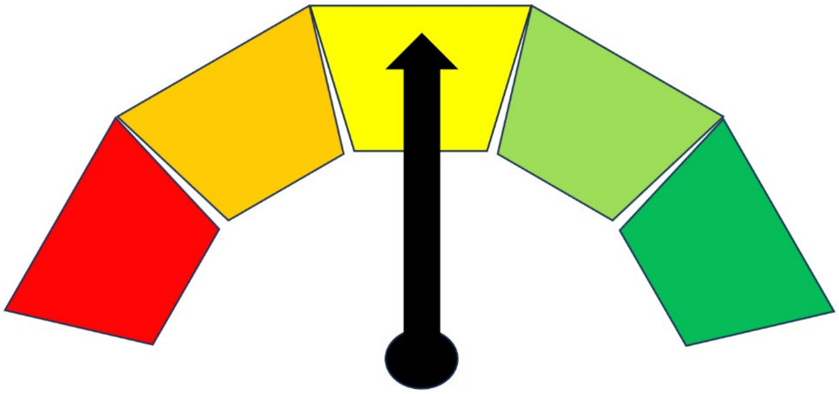

Figure S2A: Map of countries represented in study.

Graphic courtesy of mapchart.net

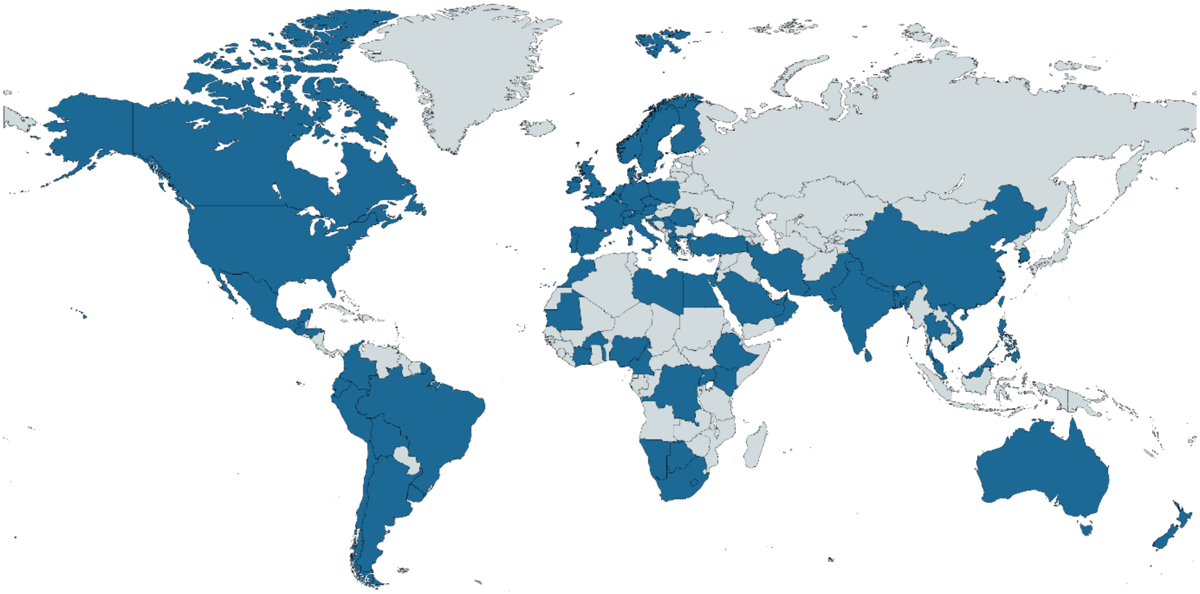

Figure. S2B. Map of countries by national participant frequency

Map of participating countries by frequency. Countries with greater numbers of participants are shaded in darker blue.

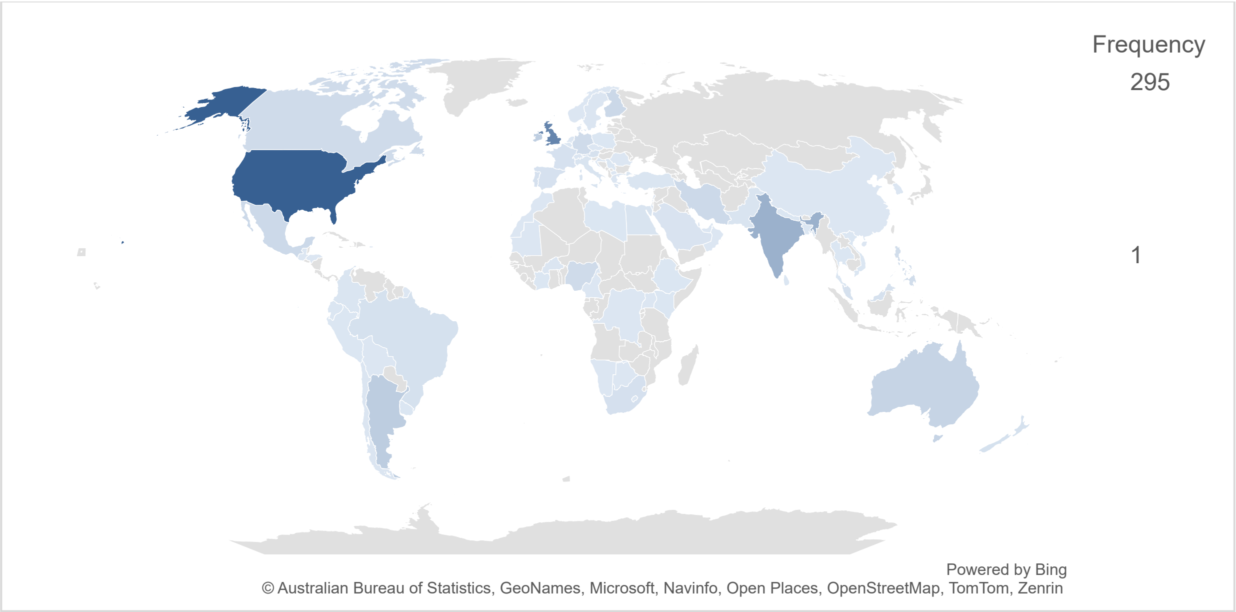

,

Figure S3A: Histogram of propensity-to-biopsy scores

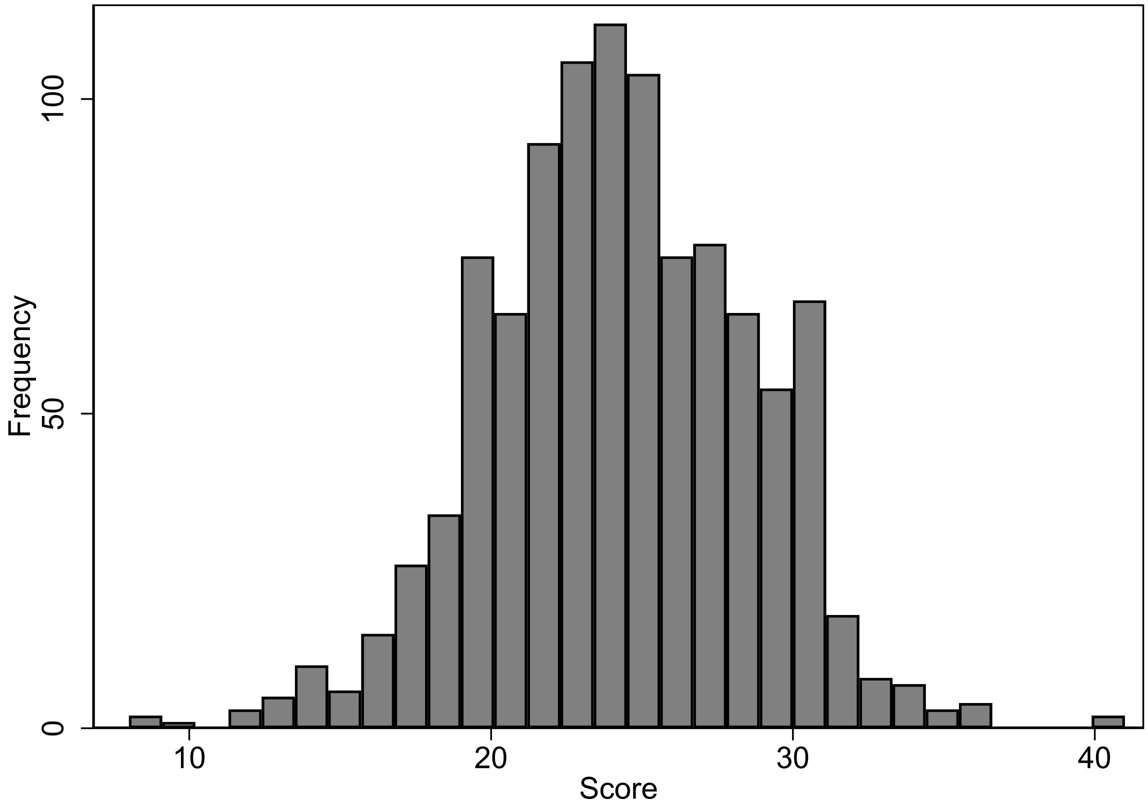

Figure S3B: Histogram of propensity-to-biopsy scores by continent

Europe

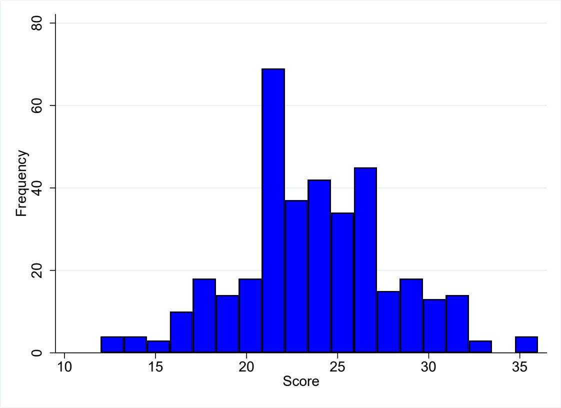

North America

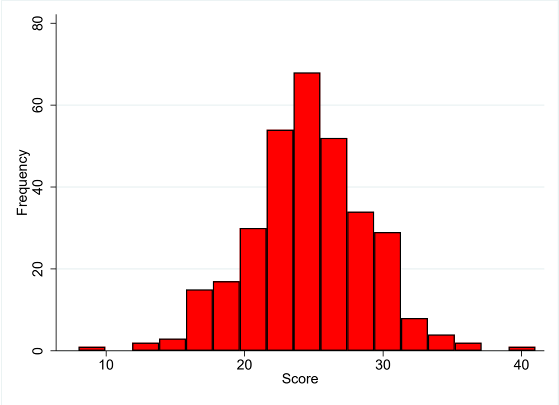

South America

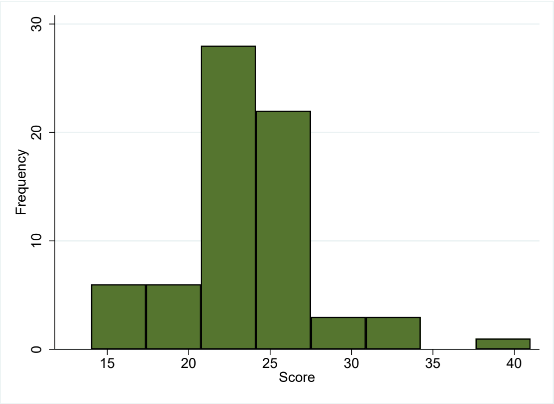

Asia

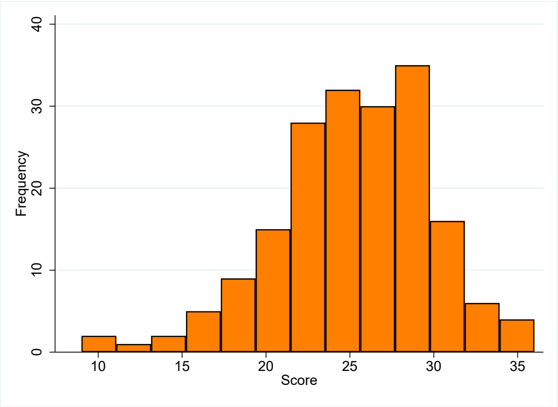

Africa

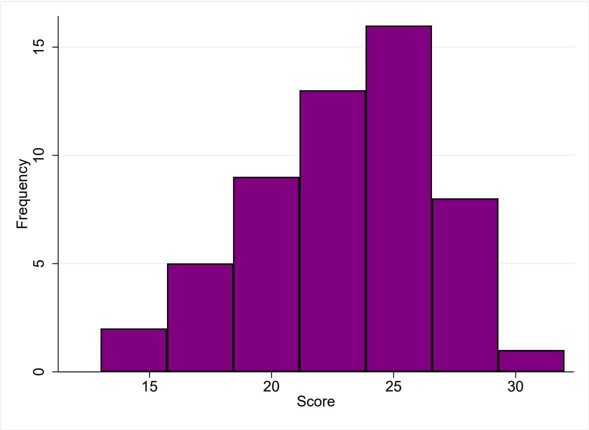

Oceana

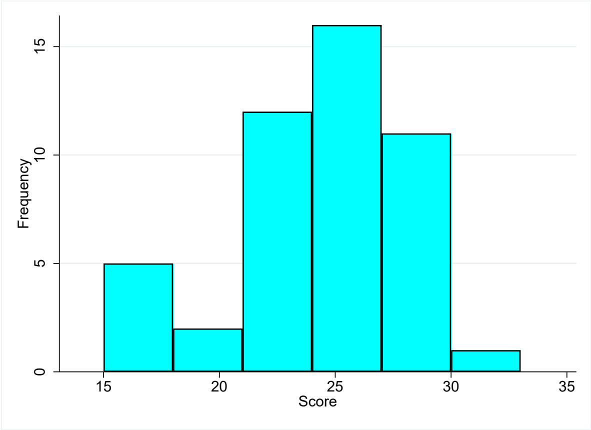

Table S1: Indications for biopsy responses

Case	eGFR	Proteinuria	Other	Definitely not	Probably not	Unsure	Probably yes	Definitely yes
1	>60	4g	Edema	7 (0.6%)	46 (4.1%)	10 (0.9%)	409 (36.3%)	655 (58.1%)
2	40	2g	Non-visible Hematuria (NVH)	3 (0.3%)	38 (3.4%)	19 (1.7%)	398 (35.6%)	659 (59.0%)
3	20	2g	NVH & normal kidney size	5 (0.5%)	58 (5.2%)	48 (4.3%)	433 (38.8%)	571 (51.2%)
4	20	2g	NVH & reduced kidney size	170 (15.3%)	571 (51.4%)	136 (12.3%)	197 17.8%)	36 (3.2%)
5	>60	0.5 to 2g	Change in one year	6 (0.5%)	109 (9.8%)	121 (10.9%)	521 (47.0%)	351 (31.7%)
6	55 to 40	0.5g	Change in one year	55 (5.0%)	398 (36.2%)	223 (20.3%)	320 (29.1%)	105 (9.5%)
7	55 to 40	0.5 to 2g	Change in one year	4 (0.4%)	72 (6.6%)	71 (6.5%)	565 (51.7%)	382 (34.9%)

Table S2: Attitudes and barriers to kidney biopsy

Statement	Global Agreement	Europe	North America	South America	Asia	Africa	Oceana
Renal biopsy helps guide future management (N=1081)	96%	94%	96%	95%	98%	99%	95%
Renal biopsy is a safe procedure (N=1080)	82%	78%	85%	81%	84%	85%	76%
Renal biopsy should be performed by a nephrologist (N=1081)	68%	68%	51%	79%	84%	85%	72%
Barriers to biopsy							
Staff availability (N=1081)	50%	46%	55%	62%	43%	56%	46%
Location/equipment availability (N=1081)	49%	42%	49%	62%	57%	64%	43%
Risks of complications (N=1081)	47%	50%	46%	46%	45%	40%	45%
Time constraints (N=1081)	44%	39%	58%	48%	33%	39%	43%

Table S3: Full list of participating countries

Country of practice	Frequency (%) (N=1179)
United States	295 (25.0)
United Kingdom	213 (18.1)
India	117 (9.9)

Argentina	56 (4.8)
Republic of Ireland	54 (4.6)
Australia	41 (3.5)
Finland	32 (2.7)
Mexico	29 (2.5)
Iran	29 (2.5)
Canada	25 (2.1)
Germany	25 (2.1)
Nigeria	25 (2.1)
Philippines	21 (1.8)
Portugal	14 (1.2)
South Africa	14 (1.2)
Brazil	13 (1.1)
New Zealand	13 (1.1)
Austria	12 (1.0)
Malaysia	12 (1.0)
France	11 (0.9)
Spain	11 (0.9)
Saudi Arabia	7 (0.6)
Egypt	6 (0.5)
Italy	6 (0.5)
Pakistan	6 (0.5)
Columbia	4 (0.3)
Taiwan	4 (0.3)
Turkey	4 (0.3)
Burkina Faso	3 (0.3)
Chile	3 (0.3)
Greece	3 (0.3)
Sweden	3 (0.3)
Switzerland	3 (0.3)
Bahrain	2 (0.2)
Bangladesh	2 (0.2)
Belgium	2 (0.2)
Ecuador	2 (0.2)
Ethiopia	2 (0.2)
Kenya	2 (0.2)
Ivory Coast	2 (0.2)
Libya	2 (0.2)
Mauritius	2 (0.2)
Nepal	2 (0.2)
Peru	2 (0.2)
Poland	2 (0.2)
Serbia	2 (0.2)
Singapore	2 (0.2)
Sri Lanka	2 (0.2)
Thailand	2 (0.2)
Uruguay	2 (0.2)
Vietnam	2 (0.2)
Aruba	1 (0.1)
Bolivia	1 (0.1)
Botswana	1 (0.1)
Cameroon	1 (0.1)
China	1 (0.1)

Croatia	1 (0.1)
Denmark	1 (0.1)
French Guiana	1 (0.1)
Guatemala	1 (0.1)
Honduras	1 (0.1)
Israel	1 (0.1)
Lebanon	1 (0.1)
Lesotho	1 (0.1)
Mauritania	1 (0.1)
Morocco	1 (0.1)
Namibia	1 (0.1)
Netherlands	1 (0.1)
North Macedonia	1 (0.1)
Norway	1 (0.1)
Oman	1 (0.1)
Puerto Rico	1 (0.1)
Qatar	1 (0.1)
Republic of the Congo	1 (0.1)
Romania	1 (0.1)
South Korea	1 (0.1)
Togo	1 (0.1)
Uganda	1 (0.1)
United Arab Emirates	1 (0.1)

Figure S4: Electronic questionnaire instrument

Start of Block: Demographic information

Consent Thank you for taking this questionnaire. You will be contributing to a PhD study of international renal biopsy practice. All responses will be anonymous. Please check eligibility criteria before continuing.

Estimated completion time: Less than 5 minutes

○ I am a medical doctor specialising in Adult Nephrology/Renal Medicine. I consent to participate

○ I do not meet the inclusion criteria and am ineligible for this study

Q2 Please verify you are a human to continue

Page Break

Q1 What age are you?

- ○ 20-29
- ○ 30-39
- ○ 40-49
- ○ 50-59
- ○ 60 or over

Q2 What is your sex?

- ○ Male
- ○ Female
- ○ Non-binary / third gender
- ○ Prefer not to say

Q3 What is your current job title?

- ○ Clinical Director or Lead
- ○ Consultant or Attending Physician
- ○ Associate Specialist or Specialty Doctor
- ○ Trainee or Fellow
- ○ Other (please specify) ____

Q4 How many renal biopsies have you performed in the last year?

- 0
- 1-5
- 5-20
- 20-50
- 50+

Page Break

Q4b When did you last perform a renal biopsy?

- ◯ Within 2 years
- ◯ Within 5 years
- ◯ More than 5 years ago
- ◯ I have never performed a renal biopsy

Q5 What is your most significant renal biopsy complication where you have been involved in any capacity?

- ◯ Death
- ◯ Nephrectomy
- ◯ Embolisation
- ◯ Blood transfusion
- ◯ Haematuria
- ◯ Other (please specify) ____
- ◯ No complications encountered

End of Block: Demographic information

Start of Block: Environment

Q1 What country do you work in?
Click box to type

▼ England ... Zimbabwe

Q2 Which sector do you work in?

- ○ Public healthcare system
- ○ Private healthcare system
- ○ Both public and private healthcare
- ○ Not sure

Q3 Where is your main place of work?

- ○ Urban hospital
- ○ Suburban hospital
- ○ Rural Hospital
- ○ Independent Clinic
- ○ Other (please specify) ____

Q4 When referred to Interventional Radiology, what is the typical waiting time for a renal biopsy?

- ○ Same day
- ○ Within one week
- ○ Within one month
- ○ Beyond one month
- ○ Unsure

Q5 In your nephrology department, who would perform the most renal biopsies?

- ○ Nephrologist
- ○ Radiologist
- ○ Nephrology trainee/fellow
- ○ Radiology trainee/fellow
- ○ Not sure

Q5b Who would most often be responsible for supervision of renal biopsies?

- ○ Nephrologist
- ○ Radiologist
- ○ Not sure

Page Break

Q6 At your institution, how long are patients observed for after an uncomplicated renal biopsy?

- ○ Less than 4 hours
- ○ 4-8 hours
- ○ 8-24 hours
- ○ Beyond 24 hours
- ○ Not sure

Page Break

6b Prior to discharge after renal biopsy, what imaging is routinely performed?

- ◯ Ultrasound
- ◯ CT scan
- ◯ MRI scan
- ◯ Other imaging
- ◯ No routine imaging

End of Block: Environment

Start of Block: Indications. V2

Q1 In your opinion- is a renal biopsy required for an **adult** in the **first detection** of an **unexplained nephrotic syndrome of proteinuria 4g/day, peripheral oedema and eGFR > 60** ml/min/1.73m2?

- ◯ Definitely yes
- ◯ Probably yes
- ◯ Unsure
- ◯ Probably not
- ◯ Definitely not

Page Break

Q2 In your opinion, is a renal biopsy required for an adult in the **first detection** of unexplained **non-visible haematuria, 2g/day of proteinuria and eGFR 40?**

- ◯ Definitely yes
- ◯ Probably yes
- ◯ Unsure
- ◯ Probably not
- ◯ Definitely not

Page Break

Q3 In your opinion, is a renal biopsy required for an adult in the **first detection** of unexplained **non-visible haematuria, 2g/day of proteinuria and eGFR 20** with **normal kidney appearances on ultrasound?**

- Definitely yes
- Probably yes
- Unsure
- Probably not
- Definitely not

Page Break

Q4 In your opinion, is a renal biopsy required for an adult in the **first detection** of unexplained **non-visible haematuria, 2g/day of proteinuria and eGFR 20** with **reduced kidney size on ultrasound?**

- ○ Definitely yes
- ○ Probably yes
- ○ Unsure
- ○ Probably not
- ○ Definitely not

Page Break

Q5 In your opinion, is a renal biopsy required for an adult with an **unexplained rise in proteinuria from 0.5 to 2g/day in one year with an eGFR > 60**

- ◯ Definitely yes
- ◯ Probably yes
- ◯ Unsure
- ◯ Probably not
- ◯ Definitely not

Page Break

Q6 In your opinion, is a renal biopsy required for an adult with an **unexplained fall in eGFR from 55 to 40 in one year with proteinuria stable at 0.5g/day?**

- ◯ Definitely yes
- ◯ Probably yes
- ◯ Unsure
- ◯ Probably not
- ◯ Definitely not

Page Break

Q7 In your opinion, is a renal biopsy required for an adult with an **unexplained fall in eGFR from 55 to 40 AND rise in proteinuria from 0.5 to 2 g/day in one year?**

- ◯ Definitely yes
- ◯ Probably yes
- ◯ Unsure
- ◯ Probably not
- ◯ Definitely not

End of Block: Indications. V2

Start of Block: Contraindications

Q1 What is the **minimum** acceptable **Haemogloblin** for native renal biopsy?

- ◯ 100 g/l
- ◯ 90 g/l
- ◯ 80 g/l
- ◯ Other (please specify) ____
- ◯ No minimum level

Q2 What is the **minimum** acceptable **Platelet count** for native renal biopsy?

- ○ 150 x 109/l
- ○ 100 x 109/l
- ○ 50 x 109/l
- ○ Other (please specify) ____________________
- ○ No minimum level

Q3 What is the **maximum** acceptable **International Normalised Ratio (INR)** for native renal biopsy?

- ○ 1.2
- ○ 1.4
- ○ 1.6
- ○ Other (please specify) ____________________
- ○ No maximum level

Q4 What is the **maximum** acceptable **Systolic Blood Pressure (SBP)** for native renal biopsy?

- ○ 140 mmHg
- ○ 160 mmHg
- ○ 180 mmHg
- ○ Other (please specify) ____________________
- ○ No maximum level

Page Break

Q5 How many **days** should each drug be **stopped for prior** to native renal biopsy?

	0	1	2	3	4	5	6	7	8	9	10
Aspirin ()											
Clopidogrel (Plavix) ()											
Direct oral anticoagulant (DOAC) ()											

Page Break

End of Block: Contraindications

Start of Block: Utility

Q1 *Please rank your opinion on the following statement:*
Renal biopsy is a safe procedure

- ○ Strongly agree
- ○ Somewhat agree
- ○ Neither agree nor disagree
- ○ Somewhat disagree
- ○ Strongly disagree

Q2 *Please rank your opinion on the following statement:*
Renal biopsy helps guide future management

- ○ Strongly agree
- ○ Somewhat agree
- ○ Neither agree nor disagree
- ○ Somewhat disagree
- ○ Strongly disagree

Q3 *Please rank your opinion on the following statement:*
Renal biopsy should be performed by a nephrologist

- ○ Strongly agree
- ○ Somewhat agree
- ○ Neither agree nor disagree
- ○ Somewhat disagree
- ○ Strongly disagree

Page Break

Q4 On a scale from 1 (Strongly Disagree) to 5 (Strongly Agree), to what degree do you feel each factor is a **barrier to renal biopsy?**

	1	2	3	4	5
Time constraints ()					
Location and equipment availability ()					
Risk of complications ()					
Staff availability ()					

End of Block: Utility
